# Cardiac adaptation to high altitude in the plateau pika (*Ochotona curzoniae*)

**DOI:** 10.1002/phy2.32

**Published:** 2013-07-18

**Authors:** Aurélien Pichon, Bai Zhenzhong, Dominique Marchant, Guoen Jin, Nicolas Voituron, Yun Haixia, Fabrice Favret, Jean-Paul Richalet, Ri-Li Ge

**Affiliations:** 1 Laboratoire “Réponses cellulaires et fonctionnelles à l'hypoxie“, Université Paris 13 Sorbonne Paris Cité UFR SMBH EA236374 rue Marcel Cachin, Bobigny, France; 2 Qinghai University Medical College, Research Centre for High Altitude Medicine, Qinghai UniversityXining, Qinghai, R. P. China

**Keywords:** Atropine, high altitude adaptation, hypoxia, isoproterenol, muscarinic receptors, ventricle hypertrophy, β-adrenergic receptors

## Abstract

The aim of this study was to assess maximal heart rate (HR) and heart morphological changes in high altitude living “plateau pikas” and rats bred at 2260 m. Rats and pikas were catheterized to measure HR (2260 m). After baseline measurements, 1 mg/kg of atropine (AT) and increasing doses of isoproterenol (IsoP) (0.1, 1, 10, and 100 μg kg) were injected into animals. Right (RV) and left ventricles (LV) were removed to calculate Fulton's ratio (LV + septum (S) to RV weights) and to assess mRNA expression level of β1- and β2-adrenoceptors, muscarinic m1 and m2 receptors, and vascular endothelial growth factor (VEGF). Resting HR was significantly lower in rats than in pikas and increased after AT injection only in rats. IsoP injection induced a significant increase in HR in rat for all doses, which was systematically greater than in pikas. In pikas HR was slightly increased only after the two highest concentrations of IsoP. Fulton's ratio was greater in rats compared with pikas but the LV + S adjusted for body weight was greater in pikas. Pikas showed lower β1-adrenoceptors and muscarinic m2 receptors mRNA expression but larger VEGF mRNA expression than rats both in RV and LV. These results suggest that pikas have a lower maximal HR compared with rats certainly due to a decrease in β-adrenergic and muscarinic receptors mRNA expression. However, the LV hypertrophy probably led to an increase in stroke volume to maintain cardiac output in response to the cold and hypoxic environment.

## Introduction

Long-term exposure of humans and animals to high altitude environment induces adaptative cardiopulmonary changes in order to maintain oxygen delivery to tissue under hypoxic conditions (Chiodi 1957; Ostadal and Kolar 2007). Acclimatization to hypoxia results in changes in the O_2_ transport system that appears to be directed to preserve normal levels of tissues oxygenation at rest. Prolonged exposure to high altitude hypoxia induces a decrease in chronotropic response to endogenous (exercise) or exogenous (isoproterenol; IsoP) adrenergic stimulation (Grover et al. 1986; Richalet et al. 1988, 1989). It has also been shown that increasing maximal heart rate (HR_Max_) by atrial pacing increased maximal cardiac output (CO_Max_), maximum convective O_2_ transport (TO2_Max_), and peak oxygen uptake (VO_2Peak_) of acclimatized rats (Gonzalez et al. 1998). These results supported a limiting role of HR_Max_ on VO_2Peak_ after acclimatization in this species (Favret et al. 2001) The low HR_Max_ is accompanied by a desensitization of β-adrenergic receptors in humans acclimatized to altitude (Richalet et al. 1988; Antezana et al. 1994) and a reduction in the density of myocardial β-adrenoceptors of rats exposed to prolonged hypoxia (Voelkel et al. 1981; Kacimi et al. 1992; Favret et al. 2001). Then, a possible mechanism of adaptation to hypoxia could be a desensitization of the β-adrenergic pathway in order to limit HR_Max_ allowing cardiac protection against hypoxia (Richalet et al. 1988).

The plateau pika (*Ochotona curzoniae*), a member of the *Ochotonidae* family, is a small rodent that lives in remote mountain areas at high altitude (≈5000 m). Pika fossil samples found on the north edge of the Qinghai-Tibetan plateau are about 37 million years old and this animal is considered to be fully adapted to high altitude. Therefore, the pika is a very suitable animal model for the study of adaptation mechanisms to a hypoxic environment. Previous studies about pikas (Du et al. 1984; Du and You 1992; Ge et al. 1998) have mainly examined the effects of hypoxia on the liver, neuroendocrine system, pulmonary circulation but little is known about cardiovascular adaptations. Sakai (1999) and Sakai et al. (2003) showed a lower right ventricular weight in pikas compared with sea level living rats. This difference is due to a lower pulmonary arterial pressure (PAP) and a consecutive minimal right ventricular hypertrophy. Recently Qi et al. (2008) observed that pikas could improve their adaptation to hypoxia by increasing mitochondria surface density, microvessel density, and myoglobin content in heart.

Thus, the aim of this study was to assess maximal HR in rats and in plateau pikas, and to evaluate the parasympathetic and sympathetic control of the heart in these lagomorphs by studying HR response to single dose of atropine (AT) and dose–response curves to IsoP as well as mRNA expression of β-adrenoceptors, muscarinic m1 and m2 receptors in right (RV) and left ventricles (LV). Morphological adaptations of the heart and vascular endothelial growth factor (VEGF) to long-term exposure to hypoxia were also assessed in both species.

## Materials and Methods

### Animals

Six pikas and six Wistar rats were used for this study. Pikas were captured at an altitude of 4700 m by traps in the Kekexili reserve (Hoh xil station) on Tibetan Plateau and then transported to Xining (altitude 2260 m), Qinghai, People's Republic of China, where the study was conducted from 3 to 6 days after their arrival. Animals were housed individually (to avoid fights) and were fed with Tibetan plateaus ground, grass, lichens, and additional vegetables (carrots). The experiments were not performed on the Tibetan plateau because no laboratory facilities were available at this time (electrical power, ambient temperature of 5°C…). The Wistar rats, were born and raised at Xining and were therefore moderately acclimatized to altitude. Kekexili reserve Authorities give their agreement for all procedure conducted in animals. All the experiments were carried out following the Guiding Principles in the Care and Use of Animals was done in accordance with the European council directive of 22 September 2010 (2010/63/EU).

### Pressure and HR measurements

As previously used (Ge et al. 1998), animals were anesthetized with pentobarbital sodium (30 mg/kg, intraperitonealy). The depth of anesthesia was checked periodically by paw pinch reflex responses insofar as HR and blood pressure were modified by drug injections and therefore were not good indicators. Body temperature was measured several times (Thermo Frigo; OTAX Europe, Villeurbanne, France) and maintained by means of heating lights. A polyethylene catheter (PE50) was placed in the aortic arch via the left carotid artery, and a PE10 catheter was advanced into the pulmonary artery, via the right jugular vein. Adequate placement of the catheters was established by the pressure waveform and verified at autopsy. The catheters were connected to pressure transducers (TSD104A; Biopac, Santa Barbara, CA). The pressure signal was sent to a demodulator (model DA100c, Biopac) and data were recorded by a Biopac system (MP150, BIOPAC System Inc.). PAP was recorded continuously, with mean pressures obtained by electronic integration. HR (beats per minute, bpm) was obtained directly from the systemic arterial pressure tracing.

### Atropine and IsoP dose–response curve

The HR response to a single dose of AT and to increasing doses of IsoP was determined in all anesthetized animals. After baseline HR measurement, 1 mg/kg of AT was injected to the animal. When HR was stabilized (≈3 min), IsoP was then injected into the carotid artery catheter in doses of 0.1, 1, 10, and 100 μg/kg, in 1 mL/kg of saline to obtain the dose–response curve. At the end of experiment, the heart was removed to weigh RV and LV plus septum (LV + S) to calculate Fulton's ratio (RV/[LV + S]) in order to assess right ventricular hypertrophy (Fulton et al. 1952). Results were expressed in absolute values and normalized with body weight (BW).

### Biological analyses

Semi-quantitative real time polymerase chain reaction was done for β1- and β2-adrenoceptors, muscarinic m2 receptors and VEGF mRNA expressions. Rats and pikas hearts (*n* = 6 and *n* = 9, respectively) were preserved in RNA Later (QIAGEN S.A., Courtaboeuf, France) solution and stored at −20°C for later use. Total RNA were extracted from these hearts using Tri-Reagent (Molecular Research Center, Inc, Cincinnati, OH). One microgram of total RNA was reverse transcribed using an oligo-dT20 primer and SuperScript III reverse transcriptase (Invitrogen, Cergy Pontoise, France), according to the manufacturer's instructions. Due to the lack of information about the genome of pika in databases, the primer design was based on consensus sequences obtained by multiple alignments of homologous sequences of at least five different species (5 to 12 depending to the gene studied), excepted for VEGF gene for which the pika's sequence was available in database (EU262734). Primer candidates were selected on region highly conserved (95–100%), and Basic Local Alignment Search Tool software was used to partially validate primers (Table 1). For reverse primers of muscarinic m2 receptor and β2-adrenoceptor, we were constrained to use degenerate sequences where the modified base was in the middle of the sequence. For semiquantitative PCR, the number of cycles, amount of primers, and annealing temperature were optimized. The cycle conditions were described in Table 2. The 18S ribosomal RNA was coamplified with the target genes as an internal control for comparative purposes. The cDNAs were amplified in a mixture containing 1.5 mmol/L MgCl_2_, 0.2 mmol/L of each dNTP, 0.5 Unit of recombinant Taq DNA polymerase (Invitrogen, Cergy Pontoise, France), and 5% of dimethyl sulfoxide for β1-adrenoceptors gene. Results correspond to the mean of duplicated analysis with high level of Kendall concordance (>71%).

**Table 1:** Sequences of primer used for biological analysis of rats and pikas hearts obtained by multiple alignments of homologous sequences of at least five different species

Gene		Primer sequence									Predicted PCR product size
18S ribosomal RNA	Forward primer	5′	AGG	CCA	TGA	TTA	AGA	GGG	AC	3′	153 bp
	Reverse primer	5′	TCT	GAT	CGT	CGT	CGA	ACC	TC	3′	
β1-adrenoceptor	Forward primer	5′	TGT	GCA	TCA	TGG	CCT	TCG	T	3′	∼392 bp
	Reverse primer	5′	GCA	GTA	GAT	GAT	GGG	GTT	GA	3′	
β2-adrenoceptor	Forward primer	5′	TGC	TGA	CCA	AGA	ATA	AGG	CC	3′	∼427 bp
	Reverse primer	5′	AGG	GTG	AAR	GTG	CCC	ATG	AT	3′	
Muscarinic m2 receptor	Forward primer	5′	TGT	TCA	GCT	TGG	CCT	GTG	CT	3′	∼250 bp
	Reverse primer	5′	GCA	ATC	ATC	ATR	CCT	GCC	AT	3′	
VEGF 189	Forward primer	5′	CCC	ATG	AAG	TGG	TGA	AGT	TC	3′	∼253 bp
	Reverse primer	5′	CTC	ATC	TCT	CCT	ATG	TGC	TG	3′	

For VEGF gene sequence was available in database (EU262734).

**Table 2:** PCR conditions optimized for interest genes coamplified with 18S ribosomal RNA

PCR	Primer concentration	Initial denaturation	Number of cycles	Denaturation	Annealing	Extension	Final extension
β1-adrenoceptor /18S ribosomal RNA	40 μmol/L 10 μmol/L	94°C 10 min	36	94°C 2 min 15 sec	50°C 45 sec	72°C 30 sec	72°C 7 min
β2-adrenoceptor /18S ribosomal RNA	13 μmol/L 20 μmol/L	94°C 5 min	35	94°C 1 min	55°C 1 min	72°C 1 min	72°C 7 min
Muscarinic m2 receptor /18S ribosomal RNA	21 μmol/L 17 μmol/L	94°C 5 min	33	94°C 1 min	56°C 1 min	72°C 1 min	72°C 7 min
VEGF 189 /18S ribosomal RNA	27 μmol/L 10 μmol/L	94°C 5 min	32	94°C 1 min	58°C 1 min	72°C 1 min	72°C 7 min

### Statistical analysis

Data are expressed as means ± standard deviation (SD). The normality of distribution was assessed by the Kolmogorov–Smirnov test. The effects of species (rats and pikas) and drugs (AT or IsoP) were evaluated using analysis of variance with repeated measures (ANOVA – Statistica, Stat Soft, Tulsa, OK). Newman–Keuls test was used for post-hoc test. Correlation and regression analyses were done by using the Pearson test. The Student's *t-*test was used whenever appropriate. A *P-*value <0.05 was considered as a significant difference.

## Results

### Animal characteristics

Animal characteristics were reported in Table 3. BW was significantly lower in pikas than in rats (*P* < 0.001). Pikas showed a lower RV weight adjusted for BW than rats (*P* < 0.05). However, pikas had a greater LV + S weight than rats when adjusted for BW (*P* < 0.001). There was also a greater Fulton's ratio in rats (0.44 ± 0.13) compared with pikas (0.23 ± 0.03, *P* < 0.001).

**Table 3:** Animal characteristics and heart ventricles weight in absolute values and relative to body weight

	Body weight (g)	RV weight (mg)	LV + S weight (mg)	RV/BW (mg/g)	LV + S/BW (mg/g)	Fulton ratio	PAP (mmHg)
Rats	348 ± 36	306.2 ± 36.2	717.0 ± 112.7	0.90 ± 0.23	2.05 ± 0.19	0.44 ± 0.13	21.8 ± 5.0
Pikas	133 ± 11*	88.4 ± 19.0*	375.9 ± 50.1*	0.67 ± 0.15*	2.84 ± 0.34*	0.23 ± 0.03*	13.3 ± 0.5*

Values are mean ± SD. RV, right ventricle; LV + S, left ventricle + septum; BW, body weight; PAP, pulmonary arterial pressure.

* *P* < 0.05 pikas versus rats.

Rats showed a significantly greater PAP than pikas (21.8 ± 5.0 mmHg vs. 13.3 ± 0.5 mmHg, *P* < 0.01).

### Dose–response curves

**Figure 1:**
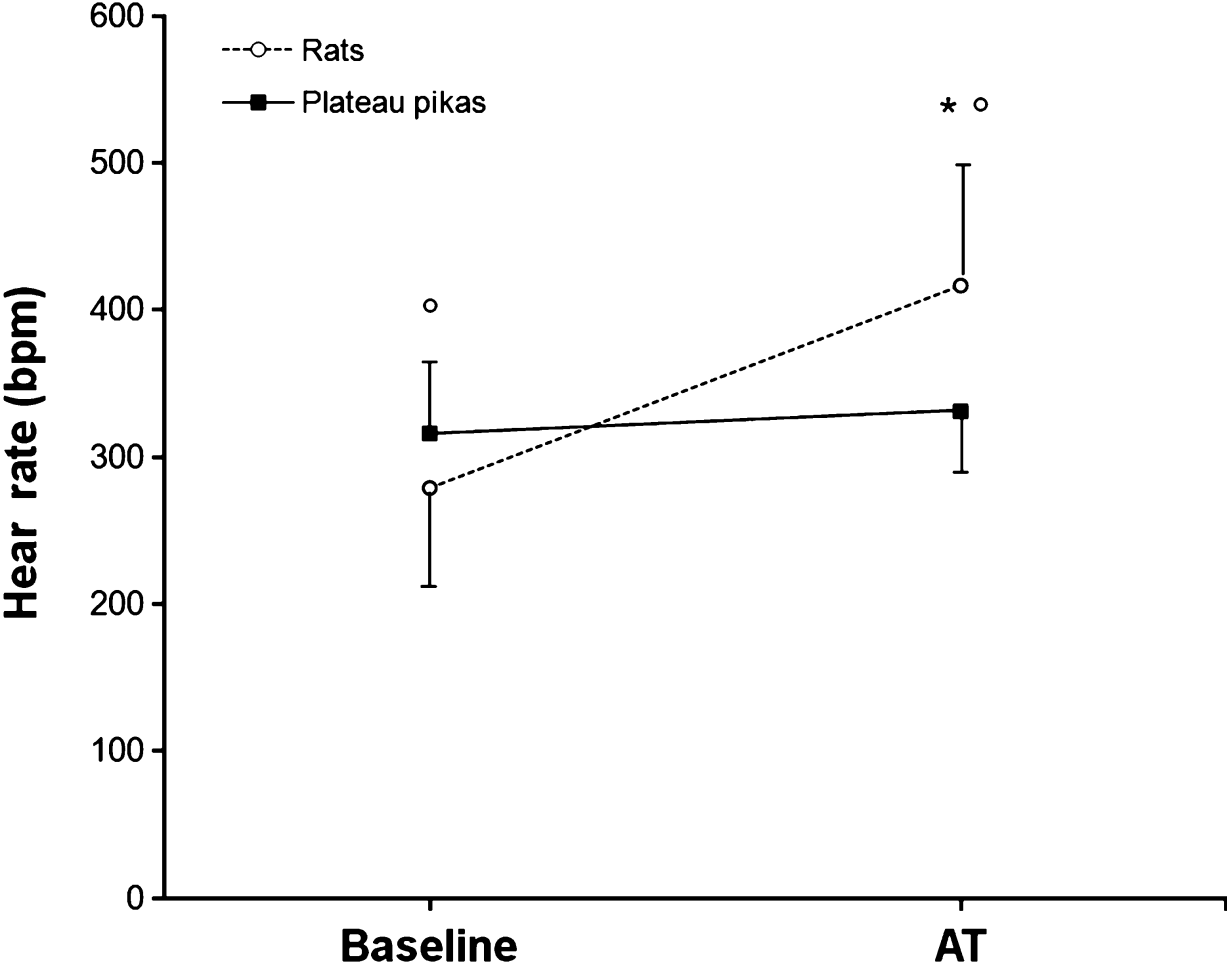
Baseline heart rate and heart rate response to a single dose of atropine (AT, 1 mg/kg) in rats and Plateau pikas. Values are mean ± SD. *, significantly different from baseline value (P < 0.05). °, significantly different from pikas value (P < 0.05).

Resting HR were significantly lower in rats (279 ± 66 bpm) than in pikas (316 ± 37 bpm). AT injection induced an increase in HR in rats but not in pikas (Fig. 1).

**Figure 2:**
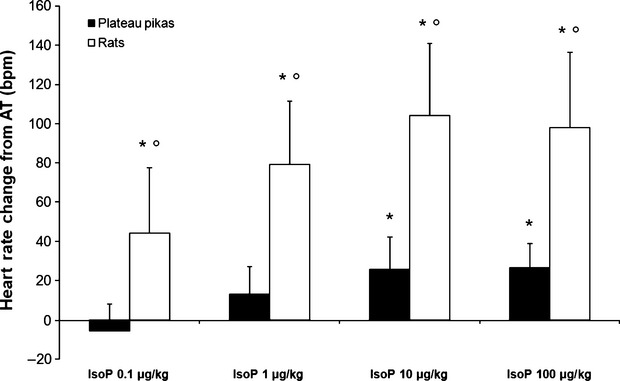
Heart rate changes after atropine injection (AT, 1 mg/kg) according to increasing doses of isoproterenol (IsoP – 0.1, 1, 10, and 100 μg/kg). Values are mean ± SD. *, significantly different from AT values (*P* < 0.05). °, significantly different from pikas value (*P* < 0.05).

In rats, IsoP injections induced a significant increase in HR from AT values for all doses (IsoP-0.1: +13%, IsoP-1: +16% bpm, IsoP-10: +20%, IsoP-100: +19%; Fig. 2). In pikas an increase in HR from AT values was observed only after IsoP-10 and IsoP-100 (+7%, *P* < 0.05). For both species the IsoP-100 did not increase HR compared with IsoP-10 and HR reach a plateau. The increase of HR was significantly greater in rats compared with pikas in every condition (*P* < 0.001).

### mRNA expression

β1-adrenoceptors mRNA expression was significantly lower in pikas than in rats, both in RV (*P* < 0.01) and LV (*P* < 0.001) (Table 4). However, the expressions of β2-adrenoceptors mRNA were significantly higher in pikas than in rats whatever the ventricle (*P* < 0.001 both). Therefore, the β1/β2-adrenoceptors ratio was lower in pikas than in rats for both ventricle (*P* < 0.001). For muscarinic m2 receptors, pikas showed a lower mRNA expression than rats whatever the ventricle (*P* < 0.001). The VEGF mRNA expression was significantly larger in pikas than in rats in both ventricles (*P* < 0.005).

**Table 4:** mRNA expression level of β1- and β2-adrenoceptors, muscarinic m2 receptors, and vascular endothelial growth factor (VEGF) expressed in relative values compared with the housekeeping gene (18S rRNA)

	Ventricle	β1-adrenoceptors	β2-adrenoceptors	β1/β2-adrenoceptors	m2 receptors	VEGF
Rats	Left	0.68 ± 0.27	0.58 ± 0.07	1.24 ± 0.59	1.08 ± 0.06	1.54 ± 0.18
	Right	0.57 ± 0.14	0.88 ± 0.21	0.57 ± 0.23	1.143 ± 0.35	1.66 ± 0.19
Pikas	Left	0.31 ± 0.09*	1.26 ± 0.22*	0.25 ± 0.09*	0.26 ± 0.11*	2.42 ± 0.22*
	Right	0.08 ± 0.10*	1.53 ± 0.22*	0.05 ± 0.07*	0.46 ± 0.19*	2.43 ± 0.33*

Values are mean ± SD.

* *P* < 0.05 pikas versus rats.

### Correlations

**Figure 3:**
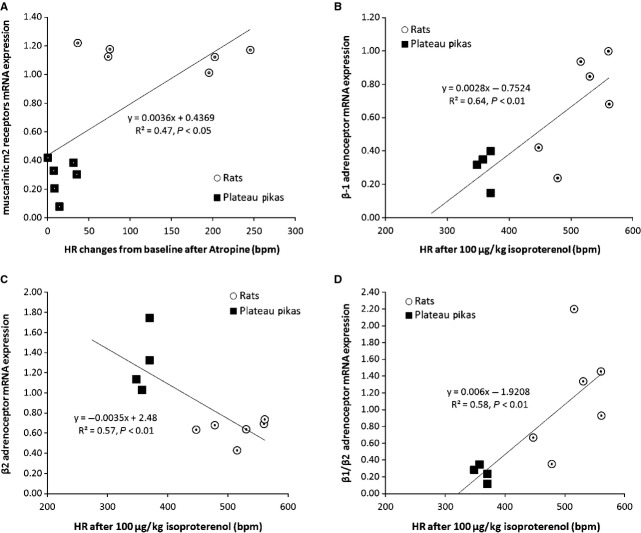
Correlations between mRNA expression and changes in heart rate (HR) after atropine (1 mg/kg) or isoproterenol (IsoP) injection (100 μg/kg) in the left ventricle of the heart Plateau Pikas and rats. Panel A. correlation between HR after atropine injection and muscarinic m2 receptor mRNA expression; Panel B. correlation between HR after IsoP injection and β1-adrenoceptor mRNA expression; Panel C. correlation between HR after IsoP injection and β2-adrenoceptor mRNA expression; Panel D. correlation between HR after isoproterenol injection and β1/β2-adrenoceptor mRNA expression.

For both species significant correlations were observed between the HR response to AT or IsoP injections and mRNA expression. The changes of HR from baseline to after AT injection were significantly and positively correlated to muscarinic m2 receptors mRNA expression for all animals (*R* = 0.68, *P* < 0.02, Fig. 3) in the left ventricle. The HR after 100 μg/Kg IsoP injection was also significantly and positively correlated to the β1-adrenoceptors mRNA expression (*R* = 0.80, *P* < 0.01 for LV and *R* = 0.69, *P* < 0.05 for RV) and to the β1/β2-adrenoceptors ratio (*R* = 0.76, P < 0.01 for LV and *R* = 0.64, *P* < 0.05 for RV). Moreover, the changes of HR from baseline after 100 μg/Kg IsoP injection were also significantly and positively correlated to the β1-adrenoceptors mRNA expression (*R* = 0.66, *P* < 0.05 for LV and *R* = 0.72, *P* < 0.01 for RV) and to the β1/β2-adrenoceptors ratio (*R* = 0.68, *P* < 0.05 for LV and *R* = 0.73, *P* < 0.01 for RV). We also observed significant correlations between β2-adrenoceptors mRNA expression in both ventricles and HR after 100 μg/Kg IsoP injection (*R* = −0.75, *P* < 0.01 for LV and *R* = −0.64, *P* < 0.05 for RV) and the changes in HR from baseline after 100 μg/Kg IsoP injection (*R* = −0.80, *P* < 0.001 for LV and *R* = −0.73, *P* < 0.01 for RV).

## Discussion

The main results of this study are that plateau pikas showed a blunted parasympathetic and sympathetic modulation of heart chronotropic function. These functional findings were reinforced by the lower mRNA expression of both β1-adrenoceptors and muscarinic m2 receptors observed in pikas hearts as compared with rats and by the significant correlations obtained between mRNA receptors and HR changes. Interestingly, pikas also exhibited a left ventricular hypertrophy as compared with rats when adjusted for BW. They also display a higher VEGF mRNA expression suggesting a greater angiogenesis of the heart via the VEGF pathway. All these data suppose a better cardiac efficiency in severe hypoxic environment due to long-term adaptation.

It has already been shown that HR_Max_ is an important limiting factor in O_2_ transport in acclimatized mammals as increasing HR_Max_ improves exercise capacity and VO_2Peak_ (Gonzalez et al. 1998). In this study, we observed a lower HR_Max_ in pikas compared with low altitude animals. A reduction of HR_Max_ after subacute hypoxia in rats (Favret et al. 2001) or chronic hypoxia in Andean guinea pigs (Leon-Velarde et al. 1996) has been linked to a decrease in β-adrenoceptor density in both heart ventricles. This reduction in β-adrenoceptors is thought to be due to prolonged agonist stimulation secondary to the increase in sympathetic drive in hypoxia (Antezana et al. 1994) and to the direct effect of hypoxia on the adrenergic receptor pathway (Voelkel et al. 1981; Kacimi et al. 1992, 1993; Webster and Bishopric 1992). In pikas, we measured a significantly lower β1-adrenoceptors mRNA expression in both ventricles, confirming the results obtained in other model of adaptation to chronic hypoxia. Higher β2-adrenoceptors mRNA expression and lower β1/β2-adrenoceptors ratio were also found. These results were consistent with the normal decrease in β1-adrenoceptors density and of the β1/β2-adrenoceptors ratio observed with chronic hypoxia. However, we cannot exclude an acclimatization effect to sea level in our adapted Plateau Pikas (4700 m) sojourning during 3–6 days at 2260 m even if this effect is probably limited as previously observed in high altitude Andean guinea pigs transported to sea level (Leon-Velarde et al. 1996).

Other studies have shown that an increase in parasympathetic tone could also account for the decrease in HR in hypoxia (Hartley et al. 1974; Hughson et al. 1994; Hopkins et al. 2003). However, we observed a very limited effect of AT injection and also a reduced muscarinic m2 receptors mRNA expression in pikas as compared with rats contrary to these previous results. This suggests that in pikas the basal parasympathetic tone is highly reduced or quite inexistent. The decrease in HR_Max_ could reduce the maximal rate of cardiac work and therefore reduce myocardial consumption, limiting the risk of myocardial ischemia (Richalet et al. 1988, 1990). Therefore, the decrease in HR_Max_ in this type of high altitude animals could be part of genetic adaptations protecting the heart from hypoxic failure.

AT and IsoP injections in pikas produce a minor increase in HR and smaller than rats bred at 2260 m. Leon-Velarde et al. (1996) also showed that Andean guinea pigs living in high altitude (4300 m) have a weaker response to IsoP compared with sea level native guinea pigs. These results have been related to a lower density of β-adrenoceptors and an increase in muscarinic m2 receptors. The small response to IsoP observed in pikas suggests a reduced density of β-adrenoceptors (Llanos et al. 2003) or an increase in muscarinic m2 receptors. It is possible that during the first period of adaptation to hypoxia, a sympathetic stimulation to the heart would have induced a down regulation of β-adrenoceptors. Subsequently, a decrease of sympathetic nervous system tone could have completed the adaptation process. It is also possible that peripheral vascular bed of pikas could respond differently as other highland species (Llanos et al. 2003) to β-adrenergic stimulation as compared with nonadapted animals (rats). However, the same relative decrease in the systemic arterial pressure was observed in rats (−2.28 ± 4.7 mmHg) and Pikas (−3.96 ± 1.45 mmHg) after IsoP injection (100 μg/Kg) suggesting that the change in HR reported between the two species was not linked to a significant change in the peripheral vasodilatory response to β-adrenergic stimulation. Leon-Velarde et al. (1998) also proposed that the prolonged stress due to the confinement of the species could be a factor for the decrement of the β-adrenoceptors density observed in rat hearts. However, pikas are wild animals without any artificial confinement. They are submitted to multiple environmental stresses associating altitude hypoxia, burrows hypoxia and hypercapnia (Kuhnen 1986) cold and low food availability.

Acclimatization to chronic hypoxia led to modifications at various degrees in the two heart ventricles (Deindl et al. 2003). In this study, we showed a differential adaptation of ventricles in pikas. We observed a lower Fulton's ratio and RV/BW value in pikas compared with acclimatized rats. However, Fulton's ratio is quite similar to see level rats while RV/BW ratio is greater (0.55 mg/g) (Favret et al. 2001) reflecting a small degree of RV hypertrophy in pikas (0.67 mg/g). On the other hand, our results provided evidence that the rats bred in Xining (low altitude) presented RV hypertrophy as assessed by their Fulton's ratio secondary to pulmonary hypertension. As pulmonary artery pressure is low in Pika, RV hypertrophy was not due to an afterload increase. It has been previously shown that pulmonary artery muscularization and hemoglobin concentration were reduced in Pikas suggesting also lower pulmonary resistance. Then, the relative RV hypertrophy in Pikas may induce a more efficient cardiac function as previously described in endurance-trained mammals (Gilbert et al. 1977). Taken together, our data and other may suggest that acclimatization is accompanied by a deleterious RV hypertrophy secondary to pulmonary hypertension while adaptation results in beneficial RV hypertrophy optimizing CO.

Concerning the left ventricle, we observed an increase in LV + S relatively to BW in pikas (+40%) compared with acclimatized rats or sea level native rats (2.19 mg/g [Favret et al. 2001]). These results suggest that pikas have a relative LV hypertrophy compared with nonadapted animals. This hypertrophy could be a way to maintain CO despite low HR_Max_ in increasing heart contractility and consequently maximal stroke volume (Favret et al. 2003; Py et al. 2005). Moreover, as the pika RV is only slightly larger than in control rats it is improbable that this small increase in RV size could explain the LV hypertrophy (Ostadal et al. 1981). Another explanation could be the direct role of cold exposure (down to −40°C) in these high altitude animals on LV hypertrophy (van Bui and Banchero 1980). Globally, the total enhancement of heart weight in pikas (RV + LV) could be compared to exercise training adaptations. Indeed, the pika, totally adapted to altitude hypoxia, shows a comparable cardiac hypertrophy with trained rats (Favret et al. 2006). This global cardiac hypertrophy is observed without any increase in pulmonary or systemic blood pressure and could be more efficient to improve CO through an increase in stroke volume than in HR. Therefore, one way of cardiac adaptation in this animal could be a slight hypertrophy and an increase in mitochondrial efficiency (Sheafor 2003) and angiogenesis as suggested by the increase in VEGF mRNA expression. These adaptations could consequently improve O_2_ uptake and utilization without increasing largely the cardiac cost, as previously suggested in Pikas on the ventilatory adaptation (Pichon et al. 2009).

## Conclusion

To conclude, the results of this study showed that plateau pikas have a smaller HR_max_ compared with rats probably due to a decrease in β-adrenergic and muscarinic receptors densities. However, the LV hypertrophy probably led to an increase in stroke volume to maintain, at least, resting CO. It will be of great interest in the future to assess the CO of this animal and its exercise capacity in order to better understand their long-term cardiovascular adaptations to hypoxia. Moreover, other animals living on the Tibetan plateaus as Tibetan antelopes could show alternative cardiovascular adaptations according to their repetitive and high intensity runs to escape predators (or poacher) in hypoxic environment.
